# Calcium silicate cements endowing bioactivity and sustaining mechanical strength of low-heat-releasing and fast-curing magnesium phosphate cements

**DOI:** 10.1093/rb/rbae100

**Published:** 2024-08-23

**Authors:** Lijun Xie, Yan Zhang, Binji Cao, Xiaoyi Jiao, Xusong Yue, Yan Xu, Xianyan Yang, Guojing Yang, Yingjie Wang, Jian Shen, Cong Wang, Xisheng Weng, Zhongru Gou

**Affiliations:** Department of Orthopaedics, The Second Affiliated Hospital, School of Medicine of Zhejiang University, Hangzhou 310009, China; Bio-nanomaterials and Regenerative Medicine Research Division, Zhejiang-California International Nanosystems Institute, Zhejiang University, Hangzhou310058, China; Department of Orthopaedics, The Second Affiliated Hospital, School of Medicine of Zhejiang University, Hangzhou 310009, China; Department of Orthopaedics, The Third Hospital Affiliated to Wenzhou Medical University & Rui’an People’s Hospital, Rui’an 325200, China; Department of Orthopaedics, The Third Hospital Affiliated to Wenzhou Medical University & Rui’an People’s Hospital, Rui’an 325200, China; Bio-nanomaterials and Regenerative Medicine Research Division, Zhejiang-California International Nanosystems Institute, Zhejiang University, Hangzhou310058, China; Bio-nanomaterials and Regenerative Medicine Research Division, Zhejiang-California International Nanosystems Institute, Zhejiang University, Hangzhou310058, China; Department of Orthopaedics, The Third Hospital Affiliated to Wenzhou Medical University & Rui’an People’s Hospital, Rui’an 325200, China; Department of Orthopedic Surgery, State Key Laboratory of Complex Severe and Rare Diseases, Peking Union Medical College Hospital, Chinese Academy of Medical Science and Peking Union Medical College, Beijing 100730, China; Department of Orthopaedics, The Second Affiliated Hospital, School of Medicine of Zhejiang University, Hangzhou 310009, China; Department of Orthopaedics, The Second Affiliated Hospital, School of Medicine of Zhejiang University, Hangzhou 310009, China; Department of Orthopedic Surgery, State Key Laboratory of Complex Severe and Rare Diseases, Peking Union Medical College Hospital, Chinese Academy of Medical Science and Peking Union Medical College, Beijing 100730, China; Bio-nanomaterials and Regenerative Medicine Research Division, Zhejiang-California International Nanosystems Institute, Zhejiang University, Hangzhou310058, China

**Keywords:** magnesium phosphate cements, calcium silicate cements, bioactivity, mechanical property, bone tissue regeneration

## Abstract

It is known that magnesium phosphate cements (MPCs) show appreciable mechanical strength and biocompatibility, but the hydration reaction processes often lead to intense heat release while the hydration products present weak resistance to mechanical decay and low bioactivity. Herein we developed an MPC-based system, which was low-heat-releasing and fast-curing in this study, by compounding with self-curing calcium silicate cements (CSCs). The MPC composed of magnesium oxide (MgO), potassium dihydrogen phosphate (KH_2_PO_4_), disodium hydrogen phosphate (Na_2_HPO_4_), magnesium hydrogen phosphate trihydrate (MgHPO_4_·3H_2_O) and chitosan were weakly basic, which would be more stable *in vivo*. The physicochemical properties indicated that the addition of CSCs could increase the final setting time while decrease the heat release. Meanwhile, the CSCs could endow MPC substrate with apatite re-mineralization reactivity, especially, which add 25 wt.% CSCs showed the most significant apatite deposition. What’s more, the mechanical evolution in buffer demonstrated CSCs could enhance and sustain the mechanical strength during degradation, and the internal constructs of cement implants could still be reconstructed by μCT analysis in rabbit femoral bone defect model *in vivo*. Particularly, appropriate CSCs adjusted the biodegradation and promoted new bone tissue regeneration *in vivo*. Totally, the MPC/CSCs composite system endows bioactivity and sustains mechanical strength of the MPC, which may be promising for expending the clinical applications of MPC-based bone cements.

## Graphical abstract

**Figure:**
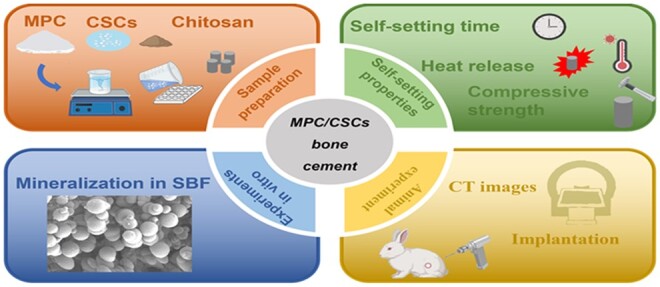

## Introduction

Because of aging, accidents, osteoporosis and other pathogenicity factors, bone fracture is common in human diseases, which endanger people’s health and cause additional medical expenses [1, 2]. When the scale of bone defect causing by orthopedic diseases exceeds the limit of human self-repair (critical bone defect), human intervention and surgical treatment are needed [3]. It is reported that nearly 4 million people in the world need to undergo bone repair surgery every year [4]. Nowadays, autografting remains the preferred solution of orthopedist for the repair of bone, while autologous bone grafting is an additional injury to patients and increases the risk of infection. Besides, for some special bone injuries, such as vertebral collapse fracture, due to anatomical factors, autologous bone transplantation is not the first choice while bone cement injection is usually used in clinical treatment. It shows that self-curing bone cement is becoming important in the clinical application of bone defects, especially in the repair of various and complex bone defects.

In the field of bone defect repair, many biomedical materials have been developed clinically based on the difference of defect site, defect degree and defect shape [5–7]. Among them, due to rheological properties of slurry, self-curing materials such as calcium phosphate cements (CPCs) can adapt to bone defects of various shapes and sizes [8], which endow them with minimally invasive repair capability for complex bone defects [9, 10]. CPCs are one of the main cements applied for bone regeneration in clinical situations since its hydration product is similar to the inorganic component of bone mineral [11]. However, CPCs tend to be with low bioactivity, poor degradation, unsatisfying injectability and mechanical strength [12].

Magnesium phosphate cement (MPC), based on its fast-curing behavior and high mechanical strength, is widely utilized in building [13]. In previous studies, it has been confirmed that some of MPC is not toxic to cells and has biocompatibility, which make it possible to be utilized as medical materials [12, 14]. It is worth mentioning that magnesium ion has multiple effects on cells [15], which can stimulate osteoblast differentiation and inhibit osteoclast formation [16]. Compared with apatite-type CPC (18–36 months [11]), MPC has a more moderate degradation efficiency (7–10 months [17]), and Mg^2+^ may be able to restrain the crystallization [18]. However, there are still drawbacks that are not clinically applicable of MPC. As to the high reaction heat and velocity of MPC [13], generally, choosing MgO sintered above 1500°C (magnesia) as the reactant and borax addition may become the effective measurements to improve the related defects [19–21], but MgO has limited effects, and borax is harmful to human body. At the same time, MPC has the phenomenon of early dissociation due to the unbalanced excess components and soluble components. On the other hand, the special skeleton-adhesive structure of MPC leads to the breakdown of the system in the later degradation process with terrible long-term structural stability [13].

It is well-known that calcium silicate cements (CSCs) mainly composed of tricalcium silicate (C3S) and dicalcium silicate (C2S) are important parts of Portland cement. In clinical application, CSCs has been utilized in pulp repair therapy [22–24]. It has been reported that CSCs could induce mineralization activity and promote cell proliferation and differentiation [25–29]. Unfortunately, CSCs tend to lead slow self-setting time and mediocre compressive strength during the entire self-setting process. Based on properties mentioned above, it is rational to assume that the addition of CSCs to MPC would endow MPC with bioactivity and probably moderate the self-setting time of the composite cements [30]. In addition, it has been studied that CSCs have volume expansion during continuous hydration, which might provide an opportunity to improve the collapse of MPC in the later stage.

In addition, the brittleness of ceramic materials is unfavorable in the process of bone tissue repair. In order to achieve good fluidity, injection and anti-collapsibility of inorganic cements, there have been many studies on the composition of organic components into inorganic cements. Many polysaccharide components such as cellulose, chitosan and chitin have been utilized to improve the plasticity and even enhance the mechanical properties of bone cements [31, 32]. Specially, it was reported recently that the mechanical strength of MPC could be greatly enhanced with a high proportion of polysaccharide content [33]. Meanwhile, organic solvents such as glycerin, polyethylene glycol may endow inorganic cements with excellent injectability [34, 35].

Based on the aforementioned consideration, it is reasonable to develop MPC-based bone cement via compounding with CSCs and chitosan. CSCs would regulate the physicochemical and biological properties of MPC substrate, including but not limited to reducing heat release, sustaining mechanical strength and endowing bioactivity while the chitosan could improve material brittleness [36] and buffer stress concentration due to stiffness differences between MPC and CSCs. Herein, we explored the effects of CSCs content on MPC-based bone cement. The research results showed that, such an MPC-based bone cements were with great potential to bone defect regeneration and repair, which might further utilize organic matter as carrier for minimally invasive treatment.

## Materials and methods

### Reagents and materials

Reagents including calcium nitrate tetrahydrate (Ca(NO_3_)_2_·4H_2_O), potassium dihydrogen phosphate (KH_2_PO_4_), tetraethyl orthosilicate (TEOS), hydrochloric (HCl) acid and chitosan were brought from Sinopharm Chemical Reagent Co., Shanghai. Disodium hydrogen phosphate (Na_2_HPO_4_), magnesium hydrogen phosphate trihydrate (MgHPO4·3H_2_O) and tris(hydroxymethyl)aminomethane (Tris) were obtained from Aladdin, Shanghai. Magnesium oxide (MgO) was from Macklin, Shanghai. Simulated body fluid (SBF) was achieved from Yuanye Bio-Technology Co., Ltd, Shanghai. All of the reagents and materials above were utilized without prior purification.

### Preparation of powders

The MPC powders included MgO, KH_2_PO_4_, Na_2_HPO_4_ and MgHPO_4_·3H_2_O (molar ratio: Mg/*P* = 3.4, (K + Na)/*P* = 1:1; mass ratio: KH_2_PO_4_/Na_2_HPO_4_ = 1:2). MgO powders were calcinated at 1550°C for 3 h to reduce its reactivity while KH_2_PO_4_ was mixed with ethanol and ball-milled for 6 h beforehand to obtain superfine powder. CSCs were composed of C3S and C2S, which were synthesized by the sol-gel method with a respective Ca/Si ratio of 2.8 and 1.8 as described previously [37]. TEOS was hydrolyzed with 20 ml of nitric acid and 400 ml of deionized water per mole for 30 minutes. Then, calcium nitrate tetrahydrate with molar volume 3 times/2 times that of TEOS was added for continuous stirring for 3 h. The resulting gel was aged in a 60°C oven for 2 days and then transferred to a 120°C oven for drying. The C3S powders were obtained by calcinating the dry gel at 1400°C in a muffle furnace for 8 h, and the C2S powders were obtained by calcinating the dry gel at 800°C in a muffle furnace for 3 h. The C3S and C2S powders were separately mixed with anhydrous ethanol to ground with zirconia ball grounding media for 6 h to obtain the superfine powder with a particle size below 5 μm after dried, and the fineness of the sol-gel-derived C2S and C3S was smaller than the other raw materials (Supplementary Table S1).

### Preparation of composite cements

MPC without CSCs (0 wt.%) and MPC-based bone cements containing different amounts of CSCs (15 wt.%, 25 wt.%, 35 wt.%, in which the content of C2S was maintained at 5 wt.%) marked as MPC, MPC/CSC-15, MPC/CSC-25 and MPC/CSC-35 were prepared as following (2.5 wt.% chitosan were included in each group; Table 1). The four groups of composite powders were well-mixed in anhydrous ethanol. Each evenly mixed powder was modulated with deionized water for 30 s to form a slurry for test of self-setting properties and to prepare samples for evaluation *in vitro* and *in vivo* separately. The samples to test *in vitro* were prepared in plastic molds (Ø 8.70 mm × 6.35 mm) and the samples *in vivo* were achieved from pouring the slurry into a PTFE mold (Ø 6 mm × 8 mm) with their respective liquid-powder ratios (LPRs; Table 2). After 1 h, the cements were demolded and then placed in 37°C water bath with saturated wet steam for 7 days. After taking out the samples, the samples *in vitro* were dried in the oven at 37°C, and the samples *in vivo* were sterilized by high-pressure steam at 120°C for use.

**Table 1.** Compositions of different MPC/CSCs composite cements powders

	MPC	MPC/CSC-15	MPC/CSC-25	MPC/CSC-35
MPC (wt.%)	97.5	82.5	72.5	62.5
C3S (wt.%)	0	10	20	30
C2S (wt.%)	0	5	5	5
Chitosan (wt.%)	2.5	2.5	2.5	2.5

**Table 2.** LPR Of the MPC-based bone cements

	MPC	MPC/CSC-15	MPC/CSC-25	MPC/CSC-35
LPR (ml/g)	0.28	0.31	0.33	0.35

### Primary characterization of powders and cement samples

The phases of MPC and MPC-based powders were characterized by PANalytical X’Pert PRO X-ray diffraction (XRD, Netherlands) at CuKα anode voltage of 40 kV and response current of 40 mA, with a step size of 0.026°/2*θ* and a range of 5°∼80°. Meanwhile, four kinds of MPC-based cement samples were rinsed with deionized water and then dried in an oven at 37°C after being cured at 37°C with saturated humidity for 7 days, and their chemical composition was analyzed by XRD after being crushed.

In addition, thin slices of each component sample with a thickness of 2 mm were prepared, kept in a water bath with saturated humidity at 37°C for 7 days, and dried in an oven at 37°C. After the gold spraying process, the surface morphology was observed through a field emission scanning electron microscope (FE-SEM, Gemini Sigma 300, Zeiss, Germany) at an accelerating voltage of 6 kV.

### Self-setting time measurement

The final setting time of the MPC-based bone cements (*n* = 3) was roughly measured by a vicar instrument (Luda, Shanghai, China) according to the national standard GB/T1346-2011. The falling distance of the final setting needle was less than or equal to 0.5 mm to determine that the cement had finally set. The initial and final setting time of the paste samples prepared with MgO before and after calcination at 1500°C were evaluated by using the same procedure as mentioned above.

### Heat release evaluation

The high heat release of MPC is a common problem, and hence the heat release of the MPC-based bone cement needs to be evaluated. The initial temperature and peak heat release temperature of the slurry modulated as samples *in vitro* of 2.0 g powder for each group (*n* = 3) were determined by infrared thermometer. The formula was as: Heat release = (*T* − *T*_0_)/2, where *T* and *T*_0_ represented the peak heat release temperature and the initial temperature, respectively.

### Initial mechanical strength measurement

In order to evaluate the mechanics of the MPC-based bone cement, cylindrical samples (*n* = 5) of different components were prepared by the preparation of *in vitro* test. Then they were cured in a water bath with saturated humidity at 37°C for different time points (1, 3, 7, 14, 21, 28, 35 days), and dried at 37°C for 3 days for testing. Taking compressive strength as the mechanical evaluation index, the load loading speed was set to 0.5 mm/min through the mechanical pressure testing machine test, and the calculation formula was as follows: *σ*_c_ = 4*F*_bc_/*πD*^2^, where *σ*_c_, *F*_bc_ and *D* were compressive strength, fracture stress, and sample diameter, respectively.

### Bio-dissolution *in vitro*

Taking Tris-HCl buffer (pH = 7.4, 37°C) as the soaking solution, the physicochemical behavior of the MPC-based bone cements in the soaking solution was analyzed according to the soaking liquid volume and sample mass ratio of 50 ml/g. The samples *in vitro* were placed in the soaking solution for 35 days after a 7-day 37°C saturated humidity water bath, and the pH value of the soaking solution was monitored by a pH meter at time points 0, 1, 3, 5, 7, 14, 21, 28 and 35 days, respectively. The concentration of Ca, Mg, Na, K, Si and P elements in the composite system was determined by inductively coupled plasma atomic emission spectrometry (ICP-AES) by taking 1 ml of the supernatant soaking solutions and adding the same amount of fresh Tris-HCl soaking solution at the same time (*n* = 3). Also, the changes in pH value in the aqueous medium was measured within the initial 48 h. In addition, mass loss analysis was performed at 0, 2, 4, 6, 8 and 12 weeks. 100% fresh soaking solution was changed every 2 weeks. The samples (*n* = 3) at the corresponding time points were soaked with absorbent paper and weighed, and the samples were moistened after 7 days of 37°C saturated humidity water bath as the initial mass. The mass loss of cements was calculated by the formula: Mass loss = *M*/*M*_0_ × 100%, where *M* and *M*_0_ were the quality after and before soaking, respectively. In order to evaluate the corrosion resistance and mechanical stability of the MPC-based bone cements in liquid environment, the samples were immersed in Tris-HCl buffer for 12 weeks, and the fresh buffer was replaced by 40% every two weeks. At time points 0, 2, 4, 8 and 12 weeks, the samples (*n* = 5) were taken out and dried at 37°C, and the compressive strength was determined by mechanical pressure testing machine. The parameters and calculation formulas are consistent with those in 2.7.

### Apatite mineralization capacity *in vitro*

The biomimetic hydroxyapatite (HA) re-mineralization of the cement materials was evaluated by immersing *in vitro* samples with SBF. Thin discs of the MPC-based bone cements with different proportions were prepared using a syringe mold with a diameter of 8.70 mm and a height of 2 mm. They were kept in a water bath with saturated humidity at 37°C for 7 days, and after being autoclaved at 120°C, the samples were soaked according to the surface area to SBF solution volume ratio of 0.1 cm^2^/ml. The samples were taken out two weeks later. The surface was cleaned with deionized water and anhydrous ethanol, and dried at 37°C. After gold spraying, the surface morphology and elemental analysis were performed by SEM and energy disperse spectroscopy (EDS).

### Animal model of bone defect *in vivo*

Samples *in vivo* of the MPC-based bone cements were implanted into an artificial critical defect on the rabbit femur. According to the preset experiment, 16 male New Zealand white rabbits (about 3 kg) aged 5 months were ordered to complete the femoral condyle implantation experiment. The experiment was approved by the Ethics Committee of Zhejiang University, and the ethics batch number is ZJU20230091.

The rabbits were divided into two groups, 6 for 4-week trials and 10 for 18-week trials. Before surgery, rabbits were given a dose of 1 mg/kg of sodium pentobarbital from an ear vein according to their body weight. After cutting the flesh and periosteum, a bone defect (Ø6 mm × 8 mm) was created in the femoral head with a trephine. Samples of the MPC-based bone cements were implanted and then sutured. Erythromycin ointment was applied to the wound, and penicillin was injected intramuscular for 3 days. At the designated time points of 4 and 18 weeks, the rabbits were anesthetized and killed, and the femur specimens were removed and immobilized with 4% paraformaldehyde fixative (4% PFA).

### Radiological analysis

The preliminary observation of the bone specimens was performed by an X-ray scanner (XPERT, KUBTEC Co., USA). The specimens were imaged under an X-ray with a response current of 50 mA and a voltage of 45 kV to obtain a qualitative general evaluation of the bone and material. At the same time, the condition of new bone (volume and distribution) was analyzed by a μCT system (AX2000 CT scanner, Always Imaging, Shanghai). The scans were performed along the axis of the femur at ambient temperature with a voltage of 120 kV and a response current of 100 mA. Each bone specimen was photographed for 5 min using a micro-focused X-ray source (FineTec 160 kV, Germany) with an X-ray detector (NDT 1717M; pixel size: 139 mm; number of pixels: 3072 × 3072). The continuous cross-sectional images generated by this procedure had a resolution of 15.6 mm with an exposure time of 500 ms, using a voxel size set to 14 mm in the isotropic measurement. Through AYRecon software reconstruction, the target area is set as a cylindrical area of Ø6 mm × 8 mm, and the radius direction is expanded outward by 300 μm to compensate for the drilling error. Material and new bone were distinguished and rendered according to density differences. The required new bone volume fraction (BV/TV), residual material volume fraction (RV/TV), and bone trabecular number (Tb.N) were obtained through VG Studio MAX.

### Histological progressing

The femur specimens that were immersed in the polyformaldehyde fixing solution for more than one week were dehydrated step by step with 80–100% ethanol, washed with xylene, and then embedded in polymethyl methacrylate. After three weeks of curing, the specimens were cut with a cutting machine (SP1600, Leica, Germany) to separate the small pieces of the femoral head close to the size of the implant area, and then after grinding, each femoral head specimen was reduced to eight slices. After polishing the sheet with a polisher (MP-2B), it is stained with H&E and Masson, observed under Leica optical microscope, adjusted and analyzed by a software (Oplenic_pro).

### Statistical analysis

All data were represented by mean ± standard deviation (SD), and the data were processed by One-way analysis of variance (ANOVA) in SPSS software. When the value of *P *<* *0.05, the results were considered statistically significant.

## Results

### Primary characterization of the cements

**Figure 1.**
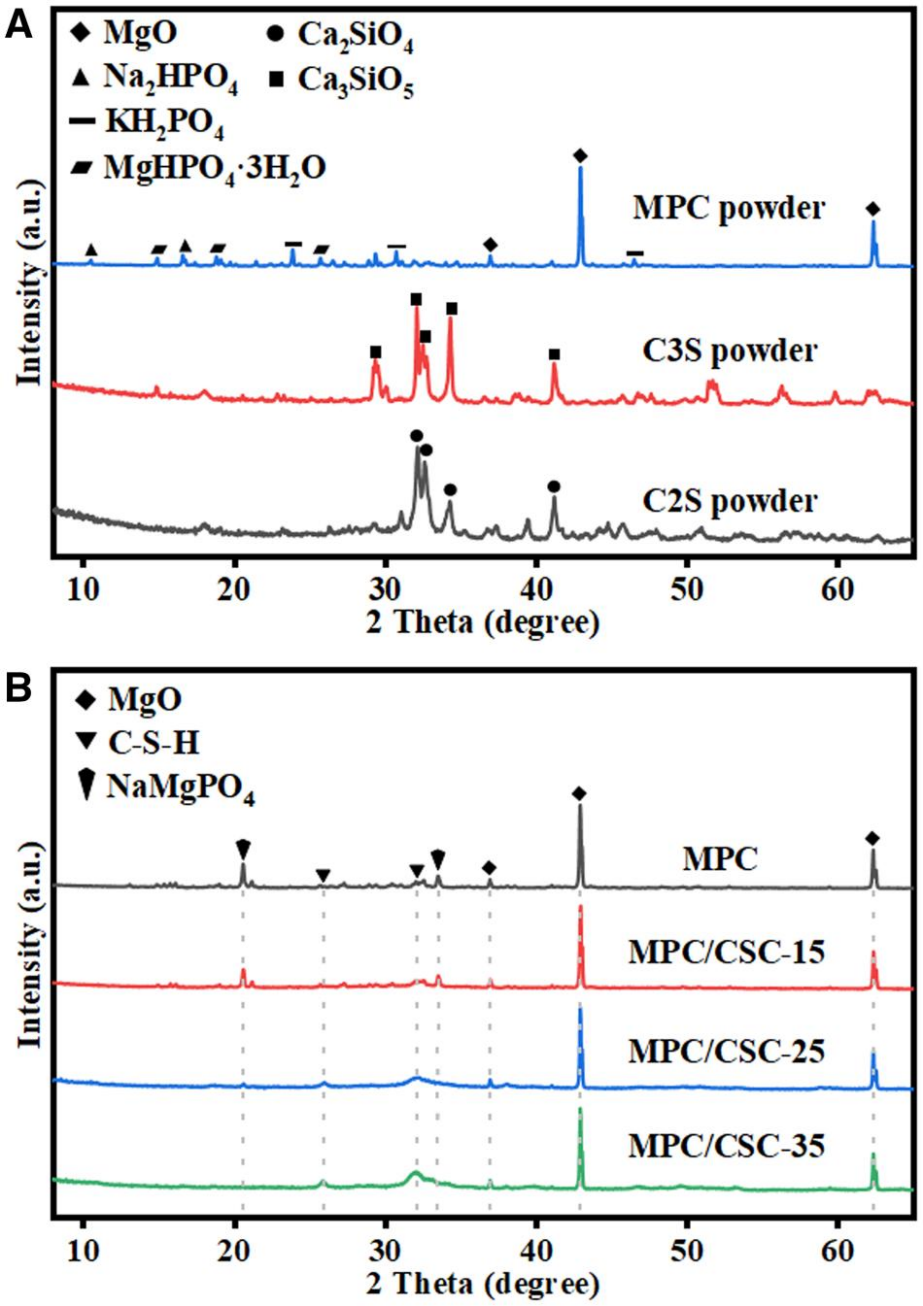
Phase composition of the precursor powders and early hydration products. XRD patterns of MPC, C3S and C2S powders (**A**). XRD images of samples with a 7-day 37°C saturated humidity water bath of MPC, MPC/CSC-15, MPC/CSC-25 and MPC/CSC-35 (**B**).

The XRD pattern (Figure 1A) for the powder of the mixed MPC contained MgO, KH_2_PO_4_, Na_2_HPO_4_ and MgHPO_4_·3H_2_O mainly showed the peeks for the MgO powder, possibly due to the high molar ratio of Mg/P in the mixtures. As for the high-temperature phase of C3S powders, due to the nonstoichiometric Ca/Si ratio (i.e. 2.8) in the sol synthetic condition, the C3S had no impurity peak of CaO at ∼37° and ∼53°/2*θ*. The sintering temperature of C2S was low and the purity was also relatively high. XRD patterns (Figure 1B) of the MPC-based bone cements after 37°C saturated humidity water bath showed that the MPC had been hydrated to produce a network of potassium magnesium phosphate (KMgPO_4_) or sodium magnesium phosphate (NaMgPO_4_) and an excess MgO skeleton. Due to the small content of KH_2_PO_4_ itself, the peak of potassium magnesium phosphate hexahydrate (KMgPO_4_·6H_2_O) visible in the XRD pattern was very low, mainly the phase of NaMgPO_4_, and a small amount of unreacted MgHPO_4_·3H_2_O. In the substrate with MgO, KMgPO_4_ and NaMgPO_4_ as the main hydration phases, the proportion of calcium silicate hydrate (CSH) gradually increased, specifically showing a steamed bun-shaped dispersion peak, while the peak of NaMgPO_4_ decreased.

**Figure 2.**
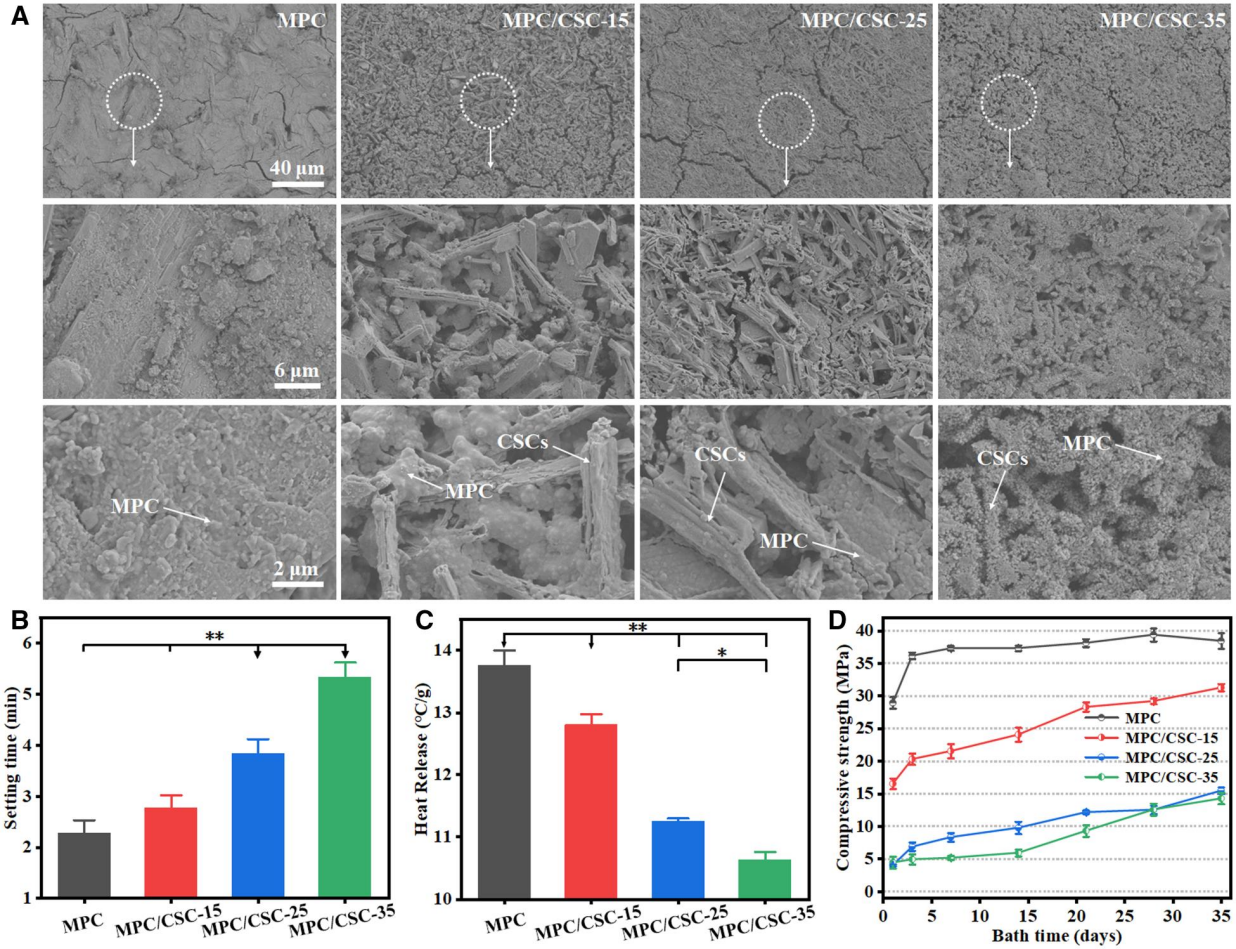
Primary structural characterization and self-setting properties of the MPC-based bone cements. SEM images of the MPC-based bone cements with a 7-day 37°C saturated humidity water bath (**A**). final setting time (**B**), heat release (**C**) of the MPC-based bone cements. Initial compressive strength of the MPC-based bone cements with a 37°C saturated humidity water bath within 35 days (**D**) (*P < 0.05, **P < 0.01).

Besides, the microstructure of MPC, MPC/CSC-15, MPC/CSC-25 and MPC/CSC-35 could be shown by SEM observation (Figure 2A). The hydrated form of pure MPC was gelatinous, which was embedded with a lot of MgO skeleton. When 10% of C3S and 5% of C2S were added, a needle-like dispersed phase appeared in the substrate, and the interphase state showed the same situation of being wrapped by KMgPO_4_/NaMgPO_4_ network phase with MgO. As the amount of C3S increased, the needle-like C3S phase increased significantly. When the amount of C3S reached 30%, the network of MPC hydrate was interrupted into discontinuous phase. From the perspective of morphology, the needle/rod had become the skeleton of the discontinuous phase.

### Self-setting property analysis

Deionized water was utilized as the blending liquid of MPC-based bone cements, the experiment showed that the LPR required by MPC was very low. LPR = 0.28 ml/g was taken as the LPR of pure MPC phase, while the LPR required by C3S and C2S slurry itself was much larger for slurry preparation, due to smaller particle size and higher specific surface area of the silicate powders in the mixtures. Therefore, 0.31, 0.33 and 0.35 ml/g were used as the LPR of the MPC/CSCs bone cements. The results of curing time (Figures 2B and Supplementary Figure S1) showed that the rapid curing of MPC greatly accelerated the curing time of the composite system, and the addition of C3S and C2S further increased the curing final solidification time and shortened the initial solidification time (both within 6 min), and in particular the high thermal pretreatment of MgO at 1500°C would prolong ∼0.8-fold of the setting time (Supplementary Figure S1). On the other hand, the MPC used in this experiment had the characteristics of low heat release. Meanwhile, the addition of C3S, C2S and chitosan also reduced the heat release of the system, making the use of MPC-based bone cements within the range of tolerance of organisms (Figure 2C). Since the actual maternity environment in the body was close to the saturated humidity water bath environment of 37°C, the cured bone cement was placed in the saturated humidity water bath environment of 37°C to monitor its strength fluctuation and analyze the maximum mechanical strength. As shown in Figure 2D, as the content of C3S increased, the time for the MPC-based bone cements to reach the maximum mechanical strength was delayed. The results of 37°C saturated humidity water bath within 5 weeks showed that the pure MPC phase had the highest mechanical strength and the fastest time to reach the maximum strength. The mechanical strength of the groups of C3S and C2S added increased steadily and was still an upward trend even after reaching 35 days.

### Degradation of cements *in vitro*

**Figure 3.**
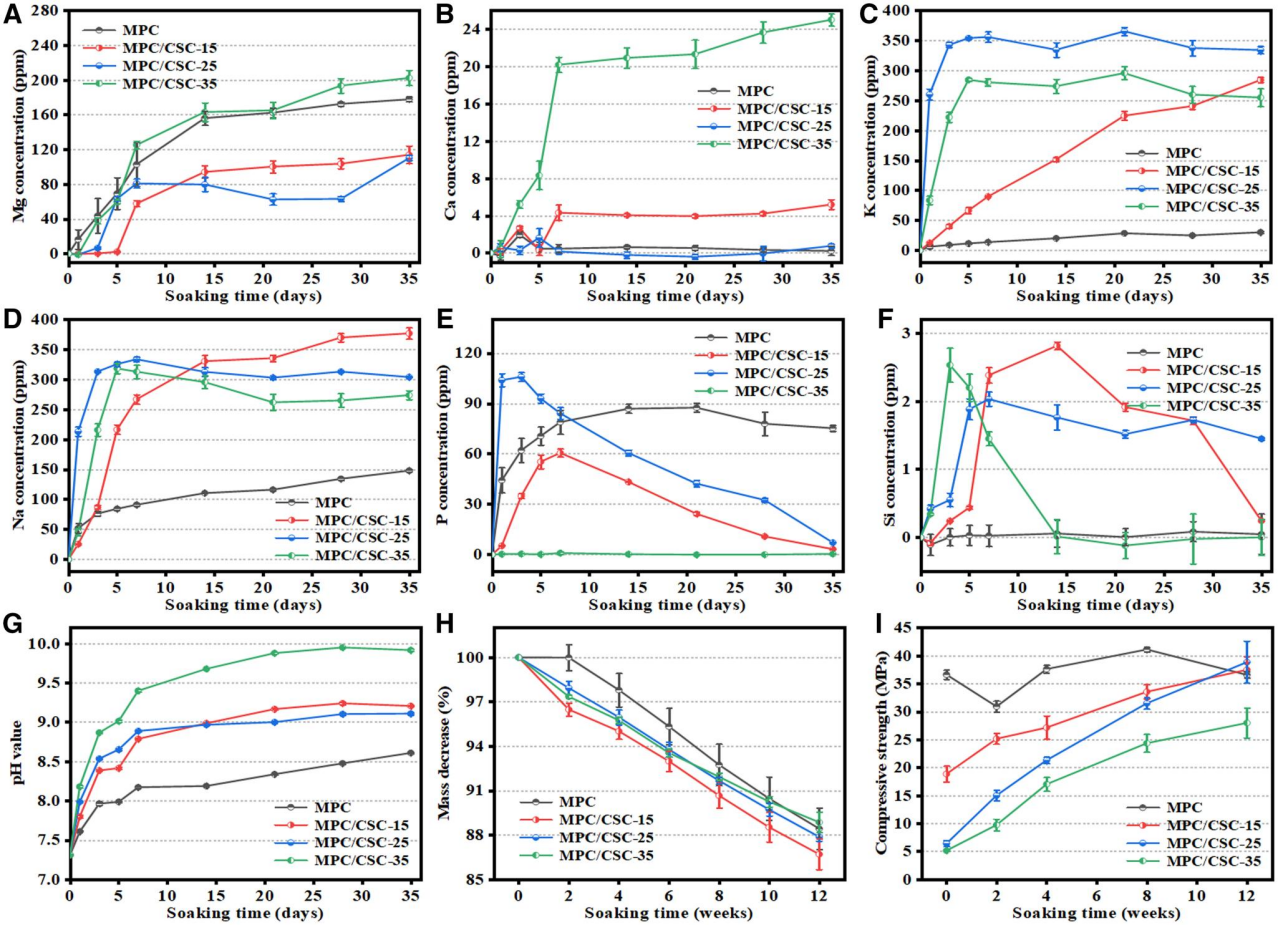
*In vitro* ion release, pH changes, mass decrease and mechanical loss in Tris-HCl buffer (pH = 7.4). Mg (**A**), Ca (**B**), K (**C**), Na (**D**), P (**E**) and Si (**F**) concentration and pH value (**G**) of *in vitro* samples (MPC, MPC/CSC-15, MPC/CSC-25, MPC/CSC-35) in Tris-HCl buffer within 35 days. Mass decrease (**H**) and mechanical loss (**I**) in Tris-HCl buffer within 12 weeks (*P < 0.05, **P < 0.01).

According to ICP analysis, the concentration of Mg ions in Tris buffer showed a steady upward trend, especially the groups of MPC and MPC/CSC-35, which were slightly faster than that of MPC/CSC-25 and MPC/CSC-15, while for the concentration of Ca ions, only MPC/CSC-35 had a significant initial increase compared with other groups. After 7 days, all groups leveled off (Figure 3A and B). The concentration of K and Na ions increased faster with more C3S before 5 days, and the curve tended to be flat after 5 days. It was worth mentioning that MPC/CSC-15 was always in a state of growth and finally exceeds all other groups, as shown in Figure 3C and D. As for phosphorus and silicon concentrations, as shown in Figure 3E and F, as the main elements in the anionic part of the system, the release level was generally low, and the release peak appeared in the early 2 weeks, and then decreased, indicating that the early release was redeposited. Among them, MPC/CSC-35 was stable as a whole, and the release speed basically does not change, and tends to zero.

The results of pH monitoring showed that the pH value of Tris buffer media soaking the MPC-based bone cements increased with the passage of time. In the early 48 h, the pH values were increased for all of cement groups (Supplementary Figure S2). And with the increase of C3S content, the pH values rose faster. It was worth mentioning that after 14 days, the pH rise in the MPC/CSC-25 began to lag behind that in the MPC/CSC-15 (Figure 3G). In general, the mass loss trend of the MPC-based bone cements was the same, and the mass of MPC itself decreased the slowest at the same time. MPC/CSC-15 increased the degradation activity of MPC with the introduction of a small amount of C3S and C2S. However, when the content of C3S continued to increase, degradation slowed again. For MPC/CSC-35, after 10 weeks, the degradation rate even lagged that of the pure MPC (Figure 3H). Similarly, the mechanical decay behavior of bone cement was crucial to evaluate its performance in use. As shown in Figure 3I, 8 weeks ago, the mechanics of MPC reached the maximum value and remained stable, while the initial strength of the groups adding C3S and C2S was lower than the pure MPC and maintaining a steady upward trend. It was clear that the MPC/CSC-15 and MPC/CSC-35 had almost the same upward slope, while the MPC/CSC-25 had a greater growth rate than both. At 12 weeks, there was a mechanical decay in the MPC, and MPC/CSC-15 just rose to the same level. The MPC/CSC-25 surpassed the MPC for the first time to reach a highest value, with a compressive strength of 43.2 MPa.

### Apatite mineralization *in vitro*

**Figure 4.**
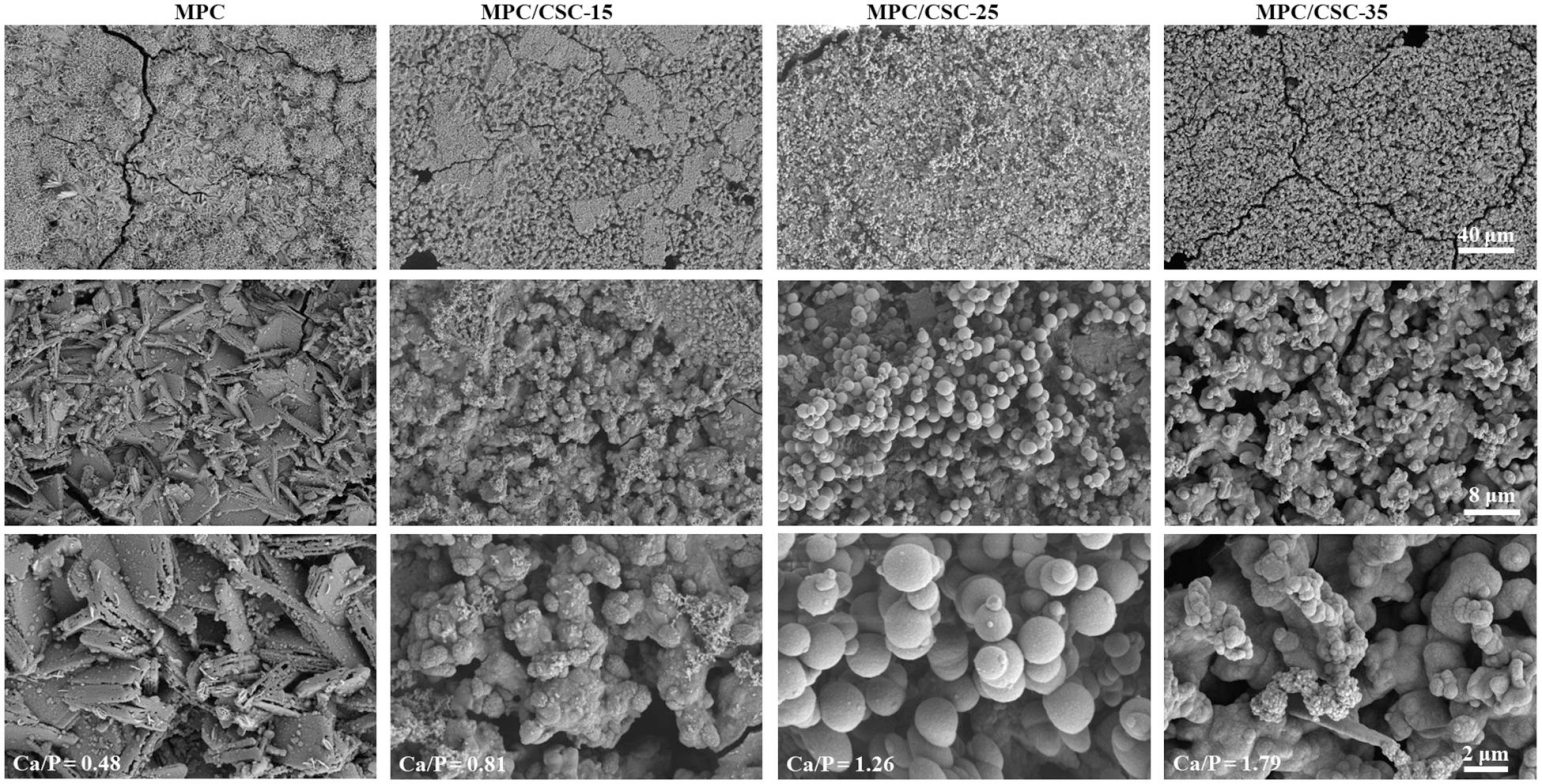
Ability to induce mineralization. SEM images of surface morphology of the MPC-based bone cements after soaking in SBF for 2 weeks.

The chemical deposition reaction (apatite re-mineralization) on the surface of MPC-based bone cements soaked in SBF was generally used to assess the remineralization ability of biological materials. Figure 4 shows the SEM image of the surface of bone cements soaked in SBF for 2 weeks. The results showed that there were no apatite microspheres on the surface of pure MPC, and very small microspheres with the diameter about 0.05 μm could be seen in MPC/CSC-15 at 8000 times field of view. With the increase content of C3S, for the group of MPC/CSC-25, the dense and full spherical apatite-like spherical deposit could be observed. Besides, with C3S further increasing to C3S-30, the average diameter of apatite spheres decreased again and the number of apatite spheres increased, developing into a mineralized surface with multinomial branches. EDS analysis showed that the overall Ca/P ratio on the surface of the microspheres in the MPC-based bone cements was 0.48, 0.81, 1.26 and 1.79, respectively. The group with the Ca/P atomic ratio of 1.26 showed the best mineralized deposition effect, and the group with the Ca/P atomic ratio of 1.79 showed a decrease but an increase in the number of mineralized microspheres. It could be seen that the surface of MPC/CSC-25 and MPC/CSC-35 were indeed a surface mineralized layer of (calcium-deficient) HA.

### Animal experiment and radiological analysis

**Figure 5.**
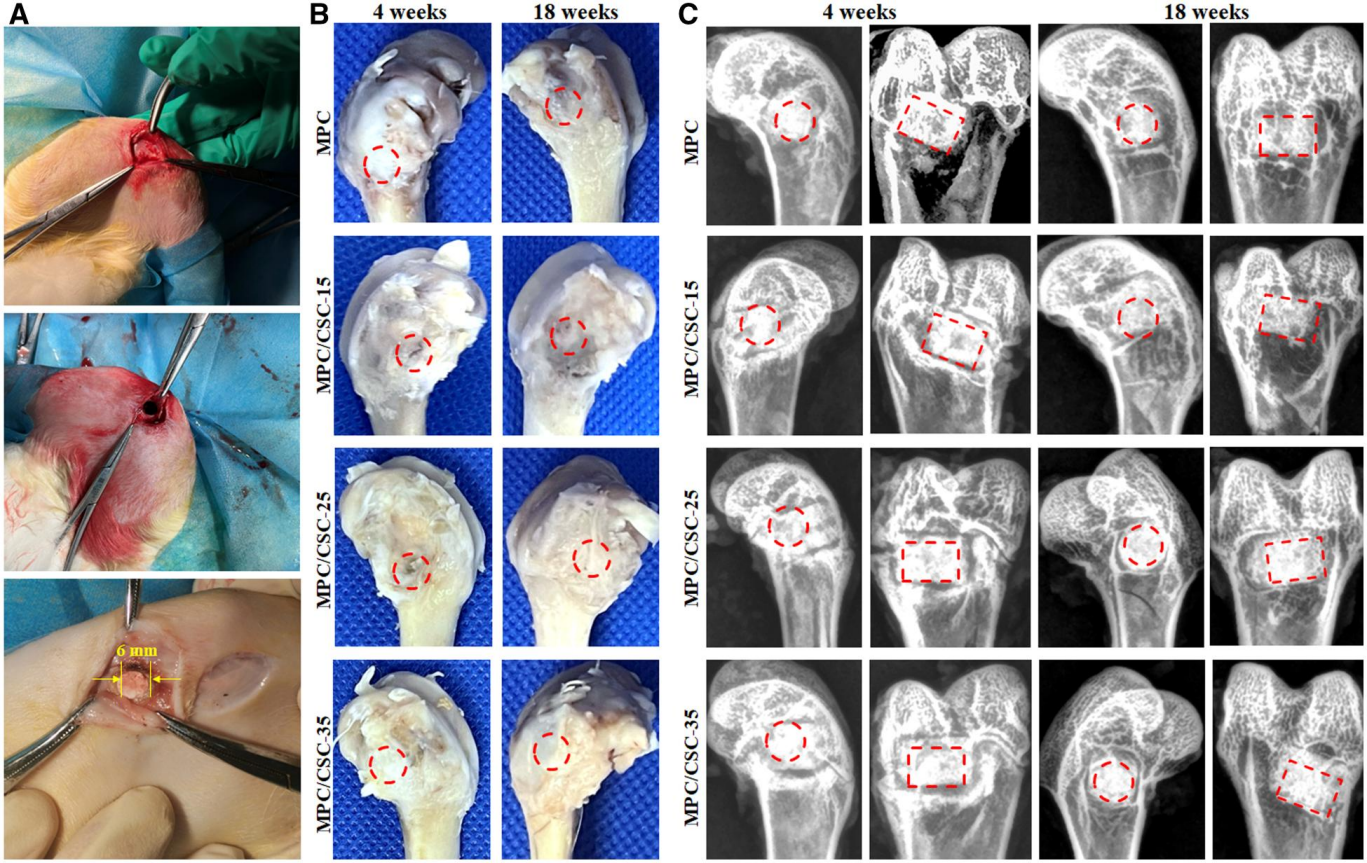
Material implantation operation process and primary evaluation of femoral bone specimens. Material implantation in the critical-sized lateral femoral defect (**A**). The specimens of the femoral bone at 4 and 18 weeks (**B**) and the corresponding images of X-ray (**C**). circle and square frame: the implanted MPC-based bone cements.

**Figure 6.**
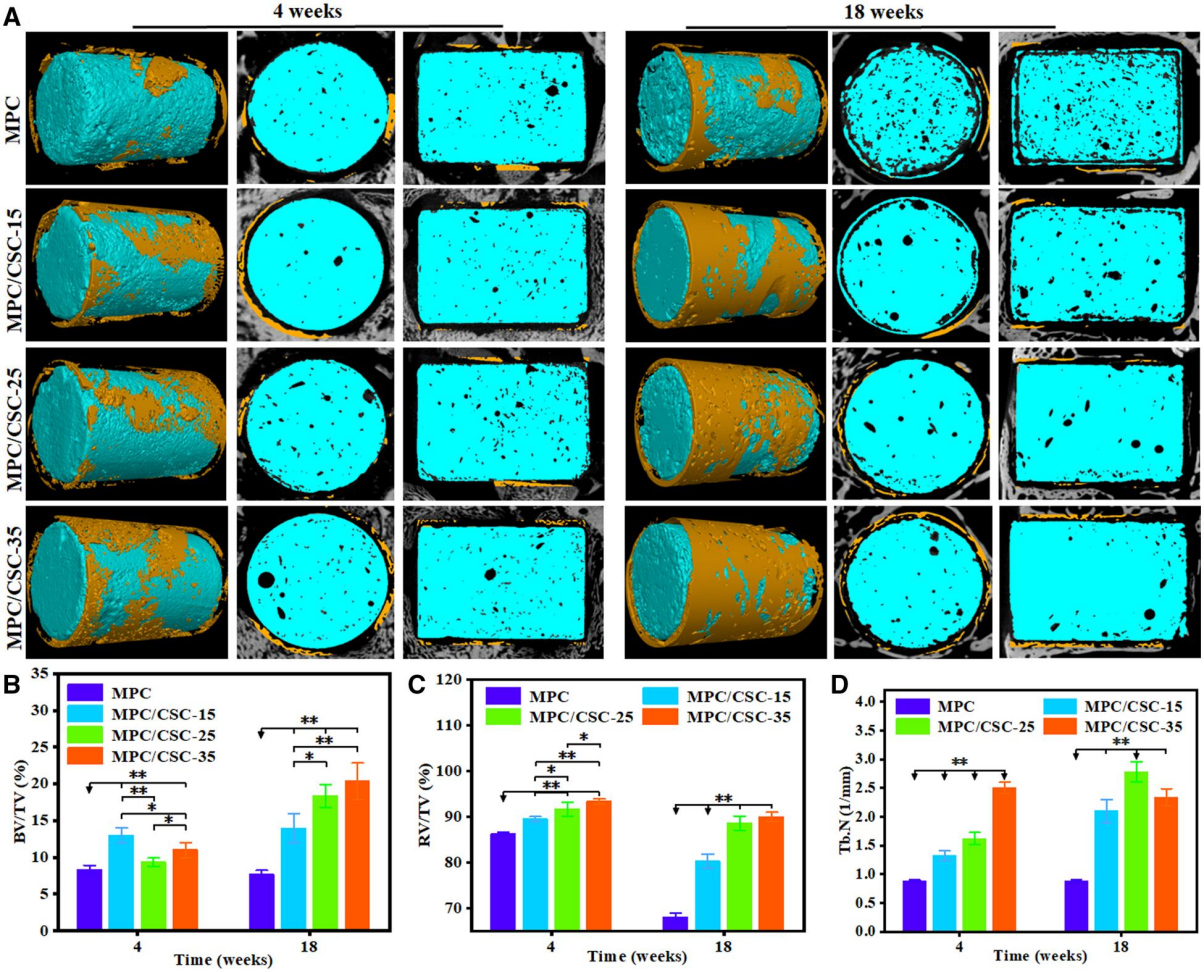
Biodegradation evaluation and osteogenic capability *in vivo*. The 3D/2D reconstructed images of micro-CT of sections at 4 and 18 weeks (**A**). Central area: the MPC-based bone cements. Peripheral area: original and new bone of 300 μm outside the cylindrical region. The quantified results of BV/TV (**B**), RV/TV (**C**) and Tb.N (**D**) at 4 and 18 weeks (*P < 0.05, **P < 0.01).

The collected specimens were scanned by X-ray to qualitatively understand the degradation of materials and the general situation of new bone regeneration. Then, 2D/3D images were scanned and reconstructed by μCT scanner, and the new bone rendering of materials was separated by density parameters. The operation process, physical specimens and corresponding X-ray scanning conditions were shown in Figure 5A–C, and the results of μCT scanning were shown in Figure 6A. At the end of the scan, the volume fraction of new bone, the volume fraction of remaining material and the number of bone trabeculae were quantitatively analyzed by the software, and the results were shown in Figure 6B–D.

The results of femur specimen and X-ray scan showed that the implant was located at the site of the bone defect. Comparing the specimens of 4 and 18 weeks, sporadic low-density areas inside the material increased in the specimens of 18 weeks, indicating that the material was degraded on the original basis. Meanwhile, there were more low-density areas inside the material in the MPC at 18 weeks than other groups. According to μCT images, the degradation degree of materials in MPC at 18 weeks was significantly increased compared with that at 4 weeks while the new bone attached to the lateral was not increased more, reflecting the poor biological activity of MPC, which was consistent with the results of *in vitro* mineralization. The addition of C3S and C2S significantly reduced the surface erosion and degradation, but significantly more bone formation near the surface.

The results of BV/TV, RV/TV and Tb.N analyzed by the software could be quantitatively found that BV/TV of four groups (MPC, MPC/CSC-15, MPC/CSC-25 and MPC/CSC-35) were 7%, 14%, 9% and 12%, respectively, at 4 weeks with little difference. The two groups of MPC and MPC/CSC-15 did not show significant increases, while others showed significant increases (Figure 6B). In addition, the characteristics of the remaining materials were consistent with the results of bone formation (BV/TV), and the degradation of the materials in MPC was close to 20%. With the contents addition of C3S and C2S, especially MPC/CSC-25 and MPC/CSC-35, the degradation of the controlled materials was only about 4% (Figure 6C). Besides, the MPC/CSC-25 groups showed a faster increase in trabecular number compared with other groups and the MPC/CSC-15 still increased compared with the MPC, while the MPC/CSC-35 showed a decrease in trabecular number at 18 weeks (Figure 6D).

### Histological assay

**Figure 7.**
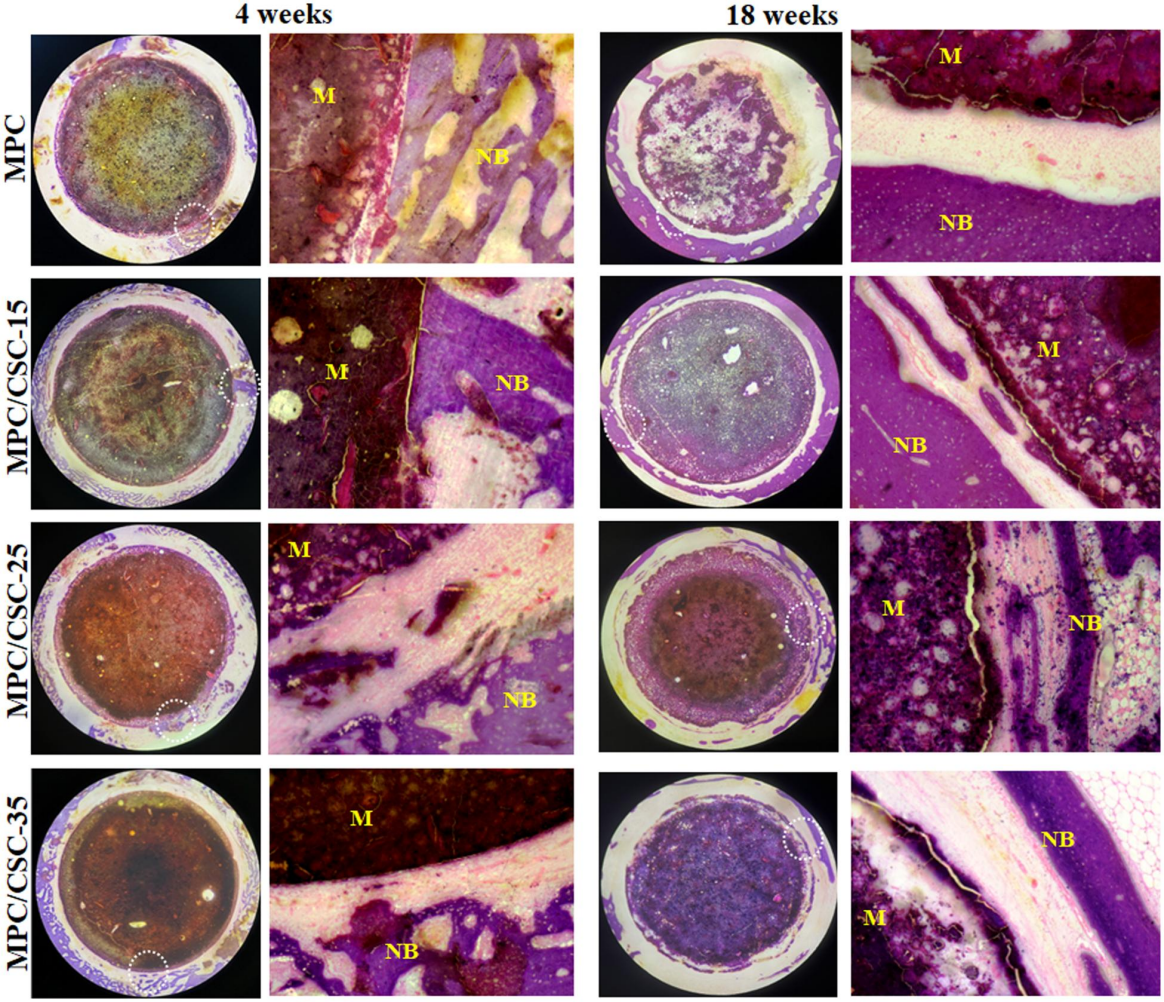
H&E staining images (30×, 100×) of the specimen sections implanted the MPC-based bone cements at 4 and 18 weeks. NB: newly formed bone. M: the MPC-based bone cements. White circle: edge of materials.

**Figure 8.**
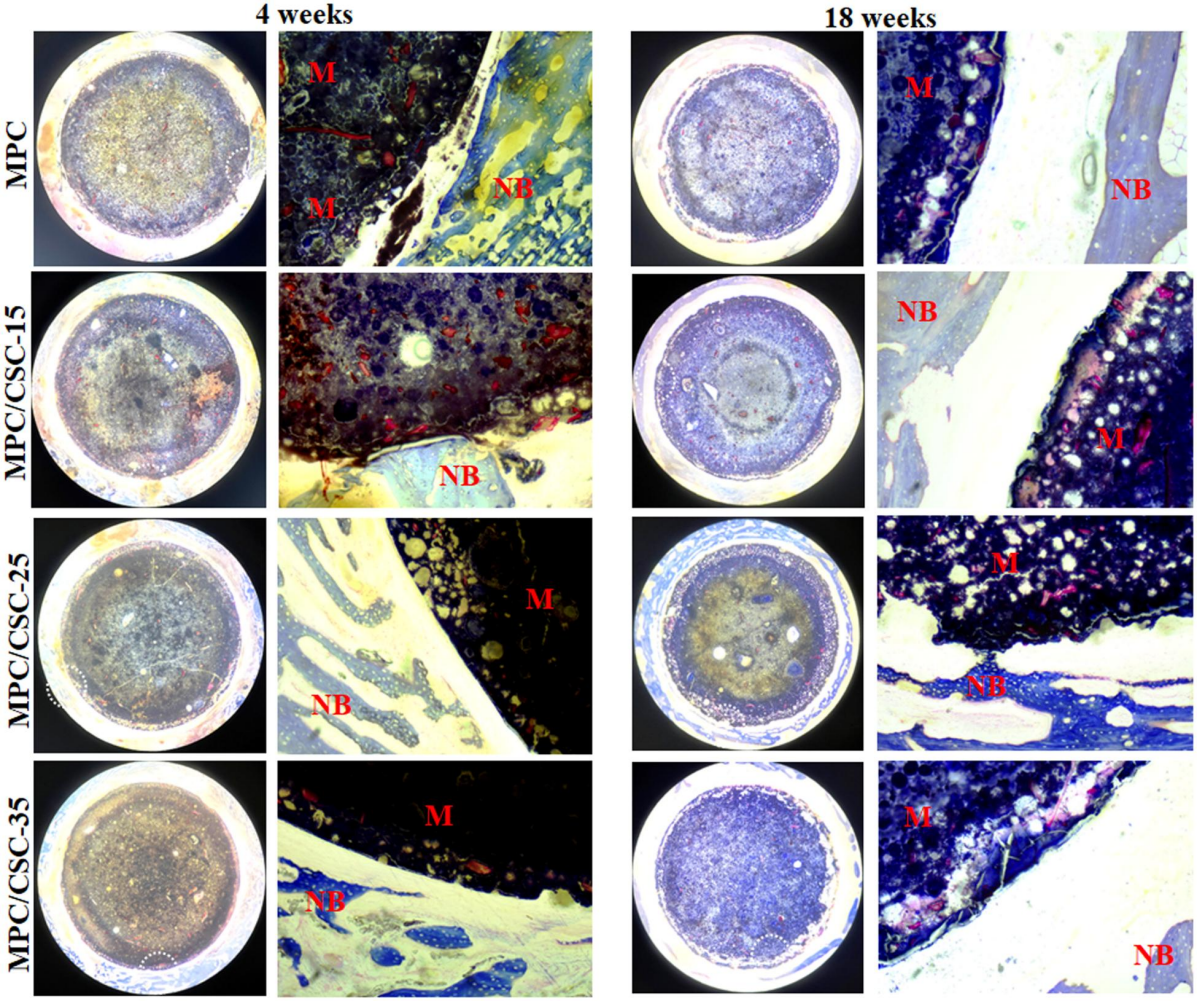
Masson staining images (30×, 100×) of the specimen sections implanted the MPC-based bone cements at 4 and 18 weeks. NB: newly formed bone. M: the MPC-based bone cements. White circle: edge of materials.

H&E and Masson staining of histological sections of rabbit femur specimens are shown in Figures 7 and 8. H&E staining showed that the MPC-based cements did not having the significant advantage of materials degrade and osteogenesis and the integration between the cement periphery and bone tissue was not ideal, supposing that excessive release of Mg^2+^ in the MPC substrate was unfavorable to bone integration. At 18 weeks, it was found that the MPC substrate was degraded into a fluffy porous state, but no bone growth was observed obviously. When the C3S and C2S were added in the modified groups, the whole material was in a relatively dense state and did not provide effective micropores for new bone to grow in, but the surrounding bone integration and the amounts of new bone increased. As for Masson staining, the group of MPC/CSC-25 and MPC/CSC-15 demonstrated the better osteogenesis at 18 weeks, surrounding by an amount of new bone, but there was still no new bone growth within the material. Besides, relatively abundant collagen fibers could be observed around the materials mixed with C3S and C2S at 18 weeks, and almost no muscle fibers were found.

## Discussion

It was found in the process of exploration that the MPC-based bone cement would degrade in the early stage due to the soluble content [38]. Besides, the solubility product of the hydrated product after solidification of MPC was larger than that of mainstream CPC materials, and its structural characteristics based on MgO as the skeleton may lead to the collapse of its cross-linked system after gradual degradation. It was inconsistent with the growth rate of new bone and thus it will lead the ability of the defect site to withstand stress to decline rapidly, resulting in repair failure. Based on this, weakly basic CSC with less soluble components was used in this study to strictly ensure the ratio of chemical reaction; C3S and C2S were used to maintain the stability of the system in the middle and later stages [39], meanwhile increasing the biological activity of the system, which will expand the possibility of clinical applications.

In construction cement, it is mostly utilized MgO and ammonium dihydrogen phosphate (NH_4_H_2_PO_4_) as precursor powders of MPCs, and the self-curing product is ‘struvite’ (NH_4_MgPO_4_·6H_2_O). Among the different self-curing product types of MPC, the NH_4_MgPO_4_ system has the highest mechanical strength. Therefore, for engineering cements, although ammonium salt will cause intense heat release, its advantages are more significant than disadvantages to some extent. Besides, the building MPC has borax and MgO with very low activity as a means to delay the heat release time, reduce the heat release and extend the curing time, and fly ash as a means to increase the mechanical stability. Unfortunately, ammonium salts decompose in the body to form ammonia, which is harmful to organisms, while borax and fly ash are also biologically toxic. Particularly, MgO with very low activity is not conducive to biodegradation and it is not conducive to enhance the bonding strength between MPC particles. Hence, MPC of this composition is not optimal for biomedical bone cement.

In order to meet the needs of clinical applications, the researchers have studied the MPC system with the same ‘struvite’ structure of KMgPO_4_·6H_2_O products. The KMgPO_4_ system has a definite chemical reaction formula and reaction product, which is similar to the reaction of NH_4_MgPO_4_ system, and is an excellent alternative material for NH_4_MgPO_4_ cement. Indeed, this system does not solve the problem of heat release and curing time, besides, the problem of mechanical decay still exists. In addition, a similar reaction was found when sodium ions replaced potassium ions in the same position. The difference is that, in mechanism, the reaction product is amorphous until it is fully hydrated. Because its reaction product NaMgPO_4_ hydrate is an indeterminate chemical formula, in this respect it is more similar to CSH. The reaction equations are as follows:

(1) $$\text{MgO}+{\text{KH}}_{2}{\text{PO}}_{4}+{5H}_{2}O={\text{KMgPO}}_{4}\cdot {6H}_{2}O$$

(2) $$\text{MgO}+{\text{NaH}}_{2}{\text{PO}}_{4}+{\text{xH}}_{2}O={\text{NaMgPO}}_{4}\cdot {\text{xH}}_{2}O$$

Based on the consideration of biosafety, the phosphate reactants of MPC series bone cement can be potassium salt and sodium salt. In addition, it is also necessary to consider the problems of high heat discharge, short curing time, and material failure. Compared with NaMgPO_4_ system, KMgPO_4_ system has a higher heat release and a longer curing time. However, under the condition of conventional MgO (referring to MgO prepared below 1500°C), the curing time is fast (<3 min), and the peak heat release is as high as 45°C/g or even more.

In the process of exploring the components of MPC, disalt hydrogen phosphate was found to significantly reduce the heat release of the system and delay the curing time. Among them, the reaction products of dipotassium hydrogen phosphate (K_2_HPO_4_) system remained in a difficult state of solidification. Besides, although the product of the reaction involving Na_2_HPO_4_ had a compressive strength of 55 MPa carrying in air under normal temperature and pressure, in the environment with water, sodium hydroxide (NaOH) was generated in the product, which would readily dissolve in water, resulting in the dissociation of the system. In order to settle the excess sodium ions in the Na_2_HPO_4_ system, the study adopted the addition of equal molar number of MgHPO_4_·3H_2_O. The results showed that MgHPO_4_ may act as an intermediate reaction product after acid-base reaction in KMgPO_4_ system, which on the one hand reduces the heat release of the system and on the other hand shorts the curing time of the system. Adding a small amount of KH_2_PO_4_ to the system of Na_2_HPO_4_ and MgHPO_4_ not only limited the curing time, but also kept the heat release in an acceptable range. The final component was to replace 1/3 mass of Na_2_HPO_4_ with KH_2_PO_4_ at a molar ratio of 3.4:1.

The weakly basic MPC in this study solved the problem of heat release, but the problem of curing time was not be solved. Considering the subsequent research works are going to solve the corresponding curing time problem with organic media as the carriers, this study more focuses on the problem of material failure and mechanical decay in the early and later stages. The alkaline component reduced the content of soluble salt and the possibility of dissolution of the early component. Besides, in order to maintain mechanical stability in the later stage, the MPC substrate was combined with different contents of C3S and C2S in this study. The resulting composite cements were based on the mechanical growth lag of C3S and C2S, which effectively maintained the mechanical strength of MPC in the later stage.

According to the XRD results (Figure 1), with the increase of C3S content, the peak of NaMgPO_4_ in the hydration products decreased after 7 days of 37°C saturated humidity water bath, and when 20% C3S was present in the MPC-based bone cement, the product showed a more obvious steamed bread-like dispersion peak. Similar to NaMgPO_4_ products, C3S, C2S and NaMgPO_4_ all had a formation process from the amorphous state to the definite crystal form. The XRD results showed that the conversion rate of NaMgPO_4_ from amorphous product to definite crystal was faster than that of C3S and C2S to definite crystal hydrated calcium silicate.

The apparent morphology of cement can help to understand its curing mechanism and strength source (Figure 2A). In general, the morphology of bone cement treated with 7 days of saturated humidity at 37°C showed that the MPC substrate was hydrated to form a gel covering the MgO skeleton and the added CSC plate/needle crystals [40, 41]. With the rapid increase of C3S content, C3S would break the cross-linked network of KMgPO_4_/NaMgPO_4_, causing damage to strength.

Because the reaction mechanism of MPCs [42, 43] was different from the hydration phase transformation of conventional CPCs [42]. The LPR concentration could be much small, which may be even 0.09 ml/g [17]. It is reasonable to assume that if the LPR maintained the same in our study, the pure MPC slurry would be too thin but CSCs slurry would be very thick. As for the MPC-based system, MPC achieved nearly no setting time increase and heat release decrease by increasing the LPR due to its acid-base reaction. Instead, the mechanical strength would decrease to a large degree. Accordingly, the LPR should be varied in each group, which is beneficial for slurry preparation and self-curing process as expected. Besides, because C3S powder has a high reaction activity, which will preferentially neutralize the reaction with hydrogen ions. Therefore, the addition of C3S further increased the reaction rate of the system, resulting in the initial setting time of the MPC-based bone cement being shortened (Supplementary Figure S2). While the hydration and hardening of bone cements was a slow process, the final setting time was extended (Figure 2B). However, due to the very short interval between the initial and final setting time of MPC substrate, it was almost negligible. When the cross-linking and hardening of MPC was occurring, the dispersion of C3S and C2S broke the cross-linking and made the system in a malleable state with poor continuity. If the stress failure occurred at this stage, the strength provided by MPC substrate would be a huge disaster. Based on the rise of pH value, the reduced activity of MgO and the slow hydration process of CSCs, the heat release was under control (Figure 2C).

In fact, it was found that when the content of C3S in the system increased, the MPC-based bone cements soaked in Tris-HCl buffer (Figure 3I) was more prone to hydration hardening than saturated humidity environment (Figure 2D). For MPC with higher MPC content and MPC/CSC-15, the intensity increase time was almost the same. The results of ion release (Figure 3A–F) show that the MPC-based bone cements were relatively negative for the release of calcium and silicon elements, among which the MPC/CSC-35 was the most special, the release of Ca^2+^ was the highest among the four groups, and the release concentration of ${\text{PO}}_{4}^{3-}$ was almost 0, showing that the high content of CSCs inhibited the degradation of MPC from the beginning. The continuous and slow increase of ${\text{PO}}_{4}^{3-}$ concentration in MPC group indicated that its degradation was not restricted when CSCs were not added. The MPC/CSC-15 and MPC/CSC-25 groups both showed the different trend of increasing first and then decreasing, indicating that ${\text{PO}}_{4}^{3-}$ were released early and then redeposited, and CSCs could stabilize the degradation of MPC about 1 month later. Moreover, ${\text{PO}}_{4}^{3-}$ release could be inhibited by high content of CSCs and promote re-mineralization due to the pH adjustment and anionic antagonism. In addition, the release of K^+^, Na^+^ and Mg^2+^ was nearly two orders of magnitude higher than other ions. The release curve of MPC/CSC-15 showed that the release curve of Na^+^ and K^+^ was more stable, while the release of Mg^2+^ was relatively delayed, indicating that a small amount of C3S could increase the degradation activity of the system, especially for the degradation of ‘struvite-like’ components, and the MgO in this study was relatively difficult to degrade. In particular, the pH of MPC/CSC-35 gradually rose above 9.5, where Mg(OH)_2_ was most readily deposited (Figure 3G), showing different dissolution characteristics. With the increase of C3S content, the initial degradation rate increased rapidly, and then tended to be stable. Both pure MPC and excessive C3S would accelerate the early release of Mg^2+^, and appropriate CSCs could better control the degradation rate of MPC system. Mass loss (Figure 3H) supported the activation of MPC/CSC-15 for early degradation. With the increase of C3S content, the longer the soaking time, the more stable the system was (Figure 3I). Besides, the increase of C3S may increase the pH of the system, thus delaying the time of complete hydration and hardening of the composite system. And then, the hydration hardening process of CSCs was accompanied by volume expansion and induces the deposition of HA [24, 44], which made up for the loss of strength caused by the reduction of density after MPC degradation. It was worth mentioning that in the 12-week immersion experiment, except for the mechanical decay of MPC group after 8 weeks, the mechanical strength of the other groups added with CSCs all increased steadily. In particular, the compressive strength of the composite system showed the fastest growth and the maximum value at 12 weeks in MPC/CSC-25. It might be that the hydrated crystals of CSCs fill the cross-linked network of MPC, and the excess MgO together forms the skeleton of the MPC-based bone cement, thus increasing the mechanical strength.

As for the biomimetic mineralization ability of the system (Figure 4), the authors found that when MPC/CSC-25 was added to the MPC system, the bone cement showed better apparent mineralization performance, which was conducive to the induction of bone formation *in vivo*. Animal femur condylar bone defect repair model confirmed that the addition of C3S and C2S could slow down the material degradation of the MPC system. By comprehensive comparison of all indexes, 15–25 wt.% calcium silicate content is more beneficial to regulate the physicochemical and biological properties of the system.

Since CSCs had the higher density than MPC, when compounded with MPC, it would present a more significant density contrast in images of X-ray (Figure 5C). According to the process of degradation *in vivo* (Figure 6A), reconstructed images of the femur specimens presented an obvious degradation of MPC at 18 weeks without CSCs. In addition, comparing Figures 7 and 8, the difference of degradation rate between the MPC skeleton and crosslinked phase was significant, which might origin from the difference of solubility product constant (*K*_sp_) of MgO, magnesium hydroxide (Mg(OH)_2_) and struvite-like products (MgO: ∼10^−25^, Mg(OH)_2_: 10^−12^∼10^−11^, NH_4_MgPO_4_: ∼10^−13^, KMgPO_4_: ∼10^−11^, NaMgPO_4_: ∼10^−10^) [45]. In fact, the rate of degradation depended mainly on the activity of MgO and the type of ‘struvite’, NaMgPO_4_ was degraded faster than KMgPO_4_, and active MgO was more easily combined with water to form Mg(OH)_2_. Therefore, the degradation structure of MPC substrate was actually able to control the preferential dissolution of its skeleton or the preferential degradation of cross-linked crystals by regulating the activity of MgO and types of phosphates. Quantitative data (Figure 6B and C) showed that MPC/CSC-15 could increase bone formation accompanied by reasonable material degradation, which still maintained the densification as well (Figure 6A). What’s more, as histological staining images presented, although the MPC/CSC-15 had the ability to promote peripheral bone formation, the poor osseous integration between material and bone defect was easily observed (Figures 7 and 8), indicating that Mg^2+^-rich environment (>20 mM) was unfavorable to osteoclastic synergistic process [46].

## Conclusion

In summary, this study determined an MPC-based composite implant by adding appropriate CSC component and ratio. The MPC-based bone cement obtained by combining CSCs with MPC of this composition has the characteristics of low heat release and expected self-curing process. Besides, the addition of CSCs will improve early corrosion and provide appreciable biological activity for the MPC-based bone cement, and also stabilize the system avoiding collapse caused by degradation in the later stage of the composite system. It is reasonable to consider that such MPC/CSCs composite bone cement may become an excellent clinical minimally invasive treatment of bone defects after improving the injection performance.

## Supplementary Material

rbae100_Supplementary_Data
