# Supplementary material for: Calcium silicate cements endowing bioactivity and sustaining mechanical strength of low-heat-releasing and fast-curing magnesium phosphate cements
Source: Regen Biomater. 2024 Aug 23;11:rbae100. doi: 10.1093/rb/rbae100 (PMC11368412; doi:10.1093/rb/rbae100)
Supplement: rbae100_Supplementary_Data [file rbae100_supplementary_data.zip › Supporiting Information.docx]

**Supporting Information (SI)**

**Table S1. Average fineness of raw materials.**

|  | MgO | KH_2_PO_4_ | Na_2_HPO_4_ | MgHPO_4_·3H_2_O | C3S | C2S |
| --- | --- | --- | --- | --- | --- | --- |
| Fineness (μm) | 42.10 | 13.51 | 227.90 | 32.11 | 4.17 | 6.67 |


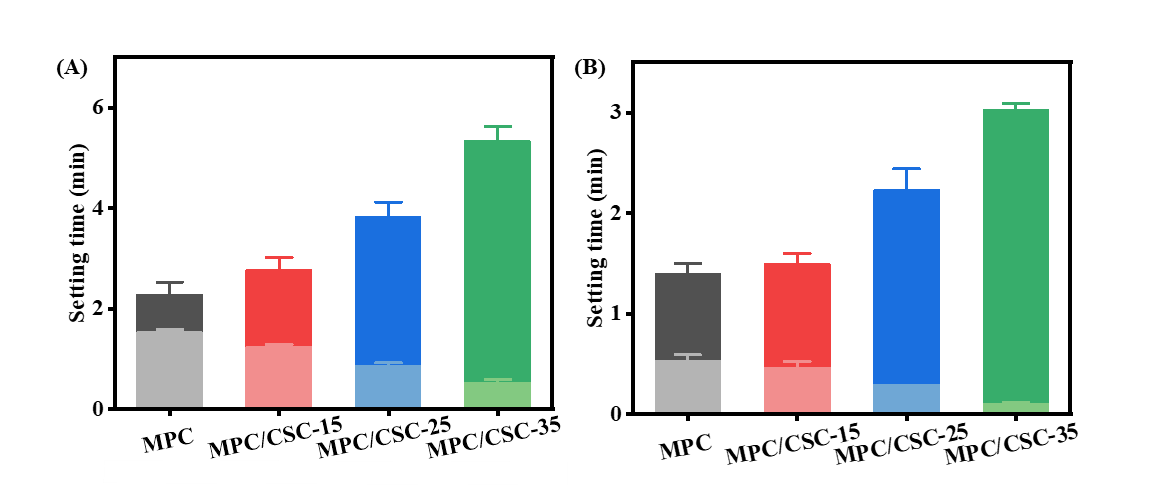


**Figure S1.** Comparison of initial and final setting time of MPC-based bone cements used with the MgO-pretreatment (A) and purchased (B).


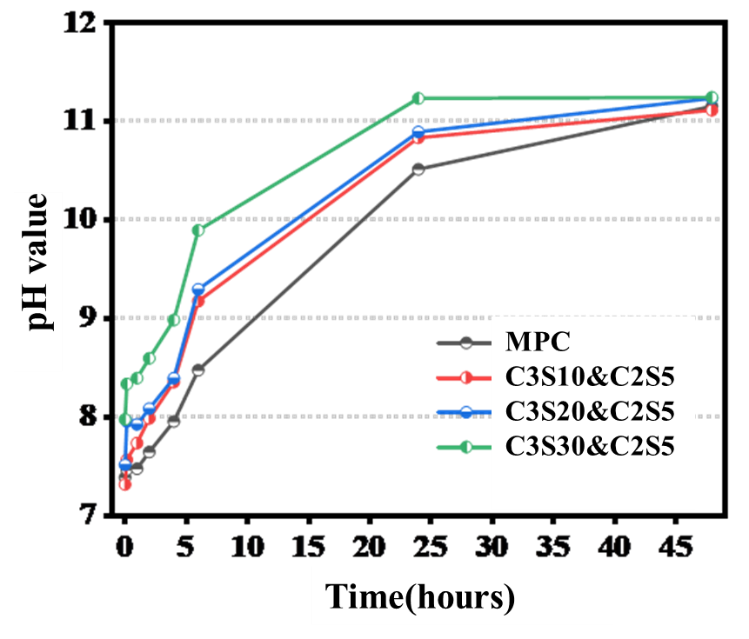


**Figure S2.** Early-stage pH value of MPC-based cements slurry (2 g / 20 mL Tris buffer) within 48 hours.
