# Supplementary material for: Calcium silicate cements endowing bioactivity and sustaining mechanical strength of low-heat-releasing and fast-curing magnesium phosphate cements
Source: Regen Biomater. 2024 Aug 23;11:rbae100. doi: 10.1093/rb/rbae100 (PMC11368412; doi:10.1093/rb/rbae100)
Supplement: rbae100_Supplementary_Data [file rbae100_supplementary_data.zip › Response to Reviewers.docx]

Dear Prof. Peppas:

Thank you for giving us the opportunity to submit a revised manuscript (Ref. RB-2024-259) entitled “***Calcium Silicate Cements Endowing Bioactivity and Sustaining Mechanical Strength of Low-heat-releasing and Fast-curing Magnesium Phosphate Cements***”. We have incorporated the format corrections. Revised portion were highlighted in blue in the revised manuscript. A list of responses to the required editorial format corrections is appended below, and we hope that the revised version of the manuscript is now acceptable for publication in your journal. We look forward to hearing from you soon.

With best wishes,

**Cong Wang, Dr.**

Department of Orthopedic Surgery, the Second Affiliated Hospital

Zhejiang University School of Medicine

Hangzhou, Zhejiang Province, 310009, China,

E-mail: [05wangcong@zju.edu.cn](mailto:05wangcong@zju.edu.cn)

**Xisheng Weng, Dr.**

Department of Orthopedic Surgery

State Key Laboratory of Complex Severe and Rare Diseases

Peking Union Medical College Hospital

Chinese Academy of Medical Science and Peking Union Medical College

Beijing 100730, China

E-mail: [xshweng@pumch.cams.cn](mailto:xshweng@pumch.cams.cn)

**Zhongru Gou, Dr.**

Bio-nanomaterials and Regenerative Medicine Research Division

Zhejiang-California International Nanosystem Institute

Zhejiang University, Hangzhou 310058, China

Tel: +86-571-8697-1539

E-mail: [zhrgou@zju.edu.cn](mailto:zhrgou@zju.edu.cn)

**Response to Reviewers**

We thank the reviewers for your positive comments, only their concerns will be addressed herein:

**Reviewer #1**

(1) The raw material information is incomplete, such as the fineness and activity of magnesium oxide used in experiments. Magnesium oxide is the main raw material of MPC, and its fineness and activity have a significant impact on the setting time and hydration heat of MPC. The manuscript prepared magnesium oxide by calcining at 1550 ℃ for 3 hours, but the magnesium oxide obtained at 1550 ℃ still has high reactivity. The manuscript did not evaluate whether the experimentally prepared magnesium oxide is suitable for preparing bone repair materials.

**Response:** Your comment is very interesting and valuable. As for the calcination pretreatment of the active MgO (purchased), it was taken into the account the too fast setting time of the slurry free of CSC components. In fact, the pure MPC could be harden within several seconds once using the purchased active MgO, and thus the pretreatment is necessary for the composite cement preparation. As you mentioned, some previous studies have investigated the high thermal pretreatment of active MgO at 1400~1800℃ has been reported in previous research. This pretreatment could inhibit some degree of reactive speed (especially the hardening reaction; see **Figure S1** in ***Supporting Information*** file) during slurry preparation/mixing process, which is suitable for implantation operation in vivo. Our pretreatment is also concerned this issue and balance the several experimental parameters such as liquid-to-solid ratio, setting time, temperature increase and so on, but not only one of parameters. Moreover, the fineness of the raw materials was listed in the new **Table S1** in ***Supporting Information*** file according to your reminding.

**Table S1. Average fineness of raw materials.**

|  | MgO | KH_2_PO_4_ | Na_2_HPO_4_ | MgHPO_4_·3H_2_O | C3S | C2S |
| --- | --- | --- | --- | --- | --- | --- |
| Fineness (μm) | 42.10 | 13.51 | 227.90 | 32.11 | 4.17 | 6.67 |

**Figure S1.** Comparison of initial and final setting time of MPC-based bone cements used with the MgO-pretreatment (A) and purchased (B).

(2) MPC is based on the acid-base reaction of magnesium oxide and phosphate to form a hardened body. Increasing the initial pH value of MPC slurry can delay the acid-base reaction of MPC, which is a basic consensus in this field. The manuscript uses calcium silicate cement to regulate the setting time and hydration heat of MPC without innovation. In addition, calcium silicate cement has a high alkalinity, and when added to the MPC system, the final alkalinity of the MPC system significantly increases, while the solubility of potassium magnesium phosphate hexahydrate, a hydration product of MPC, will significantly increase and its stability will decrease in high alkalinity environments. The manuscript did not consider the effect of adding calcium silicate cement on the alkalinity and hydration product stability of the MPC system.

**Response:** It’s a very interesting comment. According to your comment, the pH values in the Tris buffer medium during immersing the slurry in the early stage (within 48 hours) were also measured and provided in **Figure S2** in ***Supporting Information*** file. From our study, it could be seen that the pH values for the four groups of samples without and with CSC components were increased with time. In general, it might be a mis-understanding that the initial pH value of MPC slurry may delay the acid-base reaction of MPC in this system. It should be mentioned that when the reactants (H_2_PO_4_^-^ vs H_2_PO_4_^-^) are different, the reaction will be different. As for MPC system, it is a consensus that the reaction is divided into two parts: the first one is acid-base reaction, and the other one is coupling reaction. Especially, the MPC system in this article, the higher content of intermediate product MgHPO_4_ would greatly reduce the acid-base reaction and increase the proportion of the second part of the coupling reaction. Hence, the curing efficiency would increase greatly with little acid-base reaction heat release, and the acid components would prolong the curing time of the system mainly composed of MgHPO_4_ instead. In addition, the calcium silicate cements were mainly utilized to provide bioactivity and reduce the constructional collapse of the MPC substrate, which have been verified effectively from the μCT reconstruction images (see **Fig. 6**). As for the reaction speed and products, based on the little *K*_sp_ and pH of potassium/sodium magnesium phosphate hydrate, the surface re-mineralization reactivity of CSCs (see **Fig. 4**) would readily induce re-deposition of PO_4_^3-^ after some time, which could also be confirmed from the P ion release behavior in vitro (see **Fig. 3E**). Accordingly, the novelty of the present study is not only the conventional mixing mechanism, but the interactive reinforcement and compensation including self-curing reaction, bioactivity, biodegradation, and heat release, etc.

MgO + KH2PO4 + 5H2O = KMgPO4·6H2O	(1)

MgO + NaH2PO4 + xH2O = NaMgPO4·xH2O (2)

**Figure S2.** Early-stage pH value of MPC-based cements slurry (2 g / 20 mL Tris buffer) within 48 hours.

(3) The manuscript did not systematically consider the long-term stability of calcium silicate cement modified MPC. Since it is used for bone repair, systematic experiments on long-term stability should be conducted, such as simulating different human environments to study the long-term stability of calcium silicate cement modified MPC, and then conducting in vivo biological experiments.

**Response:** Your ccomment is very valuable. The study has verified the effects of bioactivity and sustaining the stability of cement construction, which is helpful to research of the further research of MPC-CSCs system. The results (mass decay and mechanical strength evolution) of Tris buffer immersion experiments in vitro for a long time stage up to 12 weeks (see **Fig. 3H,I**) and animal models (18 weeks; **Fig. 6A**) all proved the MPC-based composite system showed appreciable stability in vitro and in vivo. It is interesting that the mechanical strength was increased during immersion process, mainly because the slow hydration reaction of CSC components in comparison with the MPC component. On one hand, the low liquid system would produce a deficient water environment during the early several hours due to the consumption by MPC hydration, and thus the self-curing process led to the difficulty of water migration from the periphery environment. On the other hand, it is known that the CSC system is slow in hydration reaction; the superfine C3Si needs several hours to hydration, and C2Si would longer time stage (several days to three weeks). Therefore, the MPC-CSC composite systems could undergo a long-term increase in mechanical strength, and even the densification also increased with time, and thus the mechanical strength was increased in Tris buffer. These potential difference between MPC and CSC could contribute more valuable properties as biomedical implants.

(4) The manuscript studied the effect of calcium silicate cement content on the hydration products of MPC, but mainly focused on qualitative research and did not conduct quantitative research. For example, the XRD test results were all qualitative data. Qualitative research methods were used to prove that calcium silicate cement modified MPC can be used for bone repair, but the research methods were not rigorous enough.

**Response:** Thanks for this comment. This manuscript was mainly aimed at evaluating the effect of CSC content on the bioactivity, degradation and osteogenesis rather than only focusing on the hydration products, since the composite MPC system is a complex reaction with a lot of intermediate products in different range of hydration period itself. On the other hand, the hydration reaction is varied dynamically with time, and thus the quantitative analysis is particularly challengeable for such multi-component hybrid system. Indeed, this issue is needed to be concerned in future study.

(5) The hydration products of calcium silicate cement not only include C-S-H, but also Ca (OH) 2, which is not shown in the XRD spectrum.

**Response:** Thank you for this comment. Since CSCs were occupied a smaller proportion in the MPC-CSCs system (<35%) and the hydration time to experiments was 7 days which was not enough for CSCs to hydrate thoroughly, the XRD spectrum could not resolve the peeks to Ca(OH)_2_. In general, the peeks ascribing to Ca(OH)2 could be measured from XRD pattern for the pure C2Si or C3Si. This is mainly attribute to the appreciable free CaO content in the silicate powders by conventional sol-gel synthesis route. In our previous study (Yang X, et al. J Mater Chem B. **2014**; *2*, 3830-3838), the nonstoichiometric Ca/Si ratio in the sol system could inhibit the production of free CaO component. For instance, 1.8 of Ca/Si ratio only produce 4.8% CaO, in contrast to the 12.3% for the 2.0 of Ca/Si ratio. Thence, the minor CaO could hardly be measured by XRD analysis (see **Fig. 1A**) and thus the Ca(OH)2 also was in a very limited amount, especially in such MPC-CSC composite system.

(6) The research results of the manuscript indicate that the hydration temperature rise of MPC decreases and the setting time prolongs after adding calcium silicate cement. However, there are many studies on MPC as a bone repair material, and the manuscript has not compared with existing research results, making it difficult to evaluate the level of experimental results. In addition, the hydration temperature rise and compressive strength of MPC are related to the size of the specimen, it is difficult to evaluate the quality of the test results without comparison with existing research.

**Response:** Thank you for this suggestion. In this article, the measurement of hydration temperature rise was based on the average temperature rise of per gram powder with all of 2-gram powder. For another, the compressive strength is only decided by the basal area and force in theory, which is irrelevant to the shape. Also, the article has involved some studies on MPC. Especially, compared with Liu’s article of MPC/C3S (see Ref. 30), this article has changed the MPC powder composition with intermediate products and without borax, added chitosan to relieve cracking phenomenon and adjusted the mechanical strength in later time with C2S. Meanwhile, the article designed the animal models, which showed the results of degradation, biocompatibility and osteogenesis in vivo. All of the above have verified the initial aim at heat release reduction, bioactivity improvement and construct stability during degradation.

(7) The effect of SEM images is poor, for example, in Figure 2, when the scale is 6 μ m and 2 μ m, the hydration products of MPC and the morphology of unreacted MgO are difficult to distinguish.

**Response:** Thank you for this reminding. The MPC phase and CSCs phase have been pointed out by arrows in the revised manuscript.

(8) The manuscript also has some expression errors, such as section 2.5, GB/T 346-2001 should be replaced by GB/T 1346-2011.

**Response:** Thank you for this suggestion. The expression errors have been recorrected in the revised manuscript.

**Reviewer #2**

1) Please provide more explanation involving the difference of bioactivity between MPC and CSC.

**Response:** Your comment is very good. It was mentioned from the SEM images that MPC and MPC-CSCs presented different biomineralization microstructure, and the MPC-CSCs had more significant hydroxyapatite microsphere deposition. The difference of bioactivity between MPC and CSCs originated from the ability to induce apatite mineral deposition since the inorganic composition of bone is mainly apatite. In vivo, when Mg^2+^ concentration goes beyond 20 mM, it would be disadvantageous to cell viability. Also, Mg^2+^ itself is good for osteoblasts but not good for osteoclasts. As a result, MPC may be bioactive conditionally.

2) What means if the LPR is same in the different groups of MPC-based cements.

**Response:** Your comment is very valuable. Relative discussion has been included in the revised manuscript. The LPR choose in this article is based on the difference between MPC and CSCs and aimed at getting a higher mechanical strength while it was convenient to operate. If the LPR set as the same, the MPC would be too thin and CSCs would be too thick. As to MPC-based system, MPC achieved nearly no setting time increase and heat release decrease by increasing the LPR due to its acid-base reaction. Instead, the mechanical strength would decrease to a large degree.

3) It is valuable to explain the potential significance between initial strength decrease with increase of CSC content but the slow mechanical decay in vivo.

**Response:** Thank you for this reminding. In this base system, the CSCs have a longer hydration time than MPC and degrade much slower than MPC. As a result, the MPC-based system would provide a longer period of degradation in vivo. What’s more, CSCs have a certain volume expansion rate during hydration, which might make up for the strength loss caused by the collapse of the skeleton structure during the degradation of MPC. Meanwhile, C3S can provide mid-early strength to compensate and C2S can provide late strength to compensate.

4) Please give more discussion involving the different P and Si release behavior in vitro.

**Response:** Your comment is very interesting and high valuable. Relative discussion has been stated supplementarily in the revised manuscript. In the ion release experiments, P release of MPC increased progressively and MPC/CSC-35 was nearly zero in 4 weeks. Specially, P release of MPC/CSC-15 and MPC/CSC-25 climbed up and then declined. Si release of MPC-based system was all in single digits, and MPC/CSCs climbed up and then declined except for MPC. PO_4_^3-^ release could be inhibited by high content of CSCs and promote re-deposition of Ca-phosphate mineral due to the pH adjustment and anionic antagonism.
